# Ensuring access to life-saving medicines as countries shift from Global Fund support

**DOI:** 10.2471/BLT.19.234468

**Published:** 2019-05-01

**Authors:** Mercedes Tatay, Els Torreele

**Affiliations:** a MSF International Medical Secretary, Médecins Sans Frontières, Rue de Lausanne 78, Case Postale 1016, 1211 Geneva, Switzerland.; b Access Campaign, Médecins Sans Frontières, Geneva, Switzerland.

The Global Fund to Fight AIDS, Tuberculosis and Malaria has helped the scale-up of life-saving treatments through affordable, quality-assured medicines and diagnostics. However, faced with stagnating donor health funding,^1^ in recent years the Global Fund has revised its allocation model^2^ and its sustainability, transitions and co-financing policies.^3^

These policies are driving changes that can have negative implications for people with human immunodeficiency virus (HIV), tuberculosis or malaria. Higher prices of medicines, more use of medicines of unknown quality and more unstable supplies increase the risk of more deaths from these three preventable, treatable diseases, and exacerbate the growing global health challenge of serious drug-resistant infections.^4^ The changes also risk undermining opportunities to scale up life-saving innovations. Such innovations include the first new tuberculosis drugs in 40 years,^5^ the most effective first-line antiretroviral regimen,^6^ and a combination diagnostic platform for HIV, tuberculosis and other pathologies.^7^

The Global Fund’s revised allocation model continues to prioritize countries with the highest disease burdens and lowest incomes, but presses deprioritized countries to more rapidly mobilize alternative (especially domestic) funds to avoid reversing progress in access to life-saving medicines. Furthermore, the revised sustainability, transitions and co-financing policy requires all countries, even those with the lowest incomes, to increase their co-financing of disease programmes, including through purchasing medicines and diagnostics. Consequently, many countries shift sooner than anticipated from Global Fund-supported procurement mechanisms to national procurement processes. However, the shift to increased national procurement risks sacrificing the lower prices, quality assurance and sustainable supplies that come with Global Fund procurement.

The benefits of Global Fund-supported procurement have been achieved largely through the pooled procurement mechanism for HIV and malaria, and the Global Drug Facility for tuberculosis. These pooled, high-volume orders aggregate demand and have therefore attracted multiple suppliers offering competitive prices. In contrast, single countries need smaller volumes, which fragments demand and draws fewer suppliers or fails to interest manufacturers altogether. National procurement therefore often leaves countries with reduced negotiating power, less competition and higher prices.

Negative effects of these shifts are already apparent in some countries. From October 2016 to October 2018, 21 low- and middle-income countries paid higher prices for tuberculosis drugs and diagnostics than they would have through the Global Drug Facility procurement, while 15 countries shifting to national procurement experienced tuberculosis drug stock-outs.^8^

Another negative consequence of the Global Fund’s policy shifts relates to medicine quality. Medicines procured with Global Fund support are approved by the World Health Organization’s (WHO) pre-qualification programme or a stringent regulatory authority, such as the United States of America Food and Drug Administration. Most national procurement processes do not require WHO’s pre-qualification or regulatory authority approval, and some domestic regulatory authorities may not have the capacity to fully assess the quality and safety of medicines. Therefore, these processes introduce the risk of purchasing products of unknown quality. Between 2016 and 2018, 29 low- and middle-income countries purchased tuberculosis medicines of unknown quality and five purchased diagnostics of unknown quality.^8^

Finally, Global Fund-supported procurement circumvents the problem of pharmaceutical companies failing to register products in countries considered unattractive markets. Without the import waivers given to products procured from the Global Fund, the lack of local registration risks leading to failed tenders and lack of availability, as has already happened in some countries.

We suggest that the Global Fund, its partners and governments should undertake several steps to address this issue. The Global Fund should conduct risk and readiness assessments for countries shifting to national procurement, exempting them from such co-financing commitments if problems are identified. Donor countries should meet funding targets of the Global Fund,^9^ support affected countries in establishing strong procurement practices, and fund mechanisms that help countries optimize procurement. Countries should also revise their procurement requirements to allow the use of global and pooled mechanisms for certain life-saving products. Lastly, national tenders should adopt quality assurance requirements.

The Global Fund and WHO’s governing bodies could address these issues at their upcoming meetings in May. Their joint action is needed to avoid setbacks in scaling up affordable, quality medical tools that can prevent the deaths of people with HIV, tuberculosis or malaria.

## References

[1] Institute for Health Metrics and Evaluation (IHME) 2018 report. Seattle: Institute for Health Metrics and Evaluation; 2018. Available from: http://www.healthdata.org/sites/default/files/files/policy_report/FGH/2018/IHME_FGH_2017_fullreport_online.pdf [cited 2019 Mar 22].

[2] Overview of the allocation methodology (2014–2016): The Global Fund’s new funding model. Geneva: The Global Fund: 2014. Available from: https://www.theglobalfund.org/media/1380/fundingmodel_allocations2014-2016_methodology_en.pdf [cited 2019 Mar 22].

[3] The Global Fund sustainability, transition and co-financing Policy. Geneva: The Global Fund; 2016. Available from: https://www.theglobalfund.org/media/4221/bm35_04-sustainabilitytransitionandcofinancing_policy_en.pdf [cited 2019 Mar 18].

[4] Kranzer K, Ford N. Unstructured treatment interruption of antiretroviral therapy in clinical practice: a systematic review. Trop Med Int Health. 2011 10;16(10):1297–313. 10.1111/j.1365-3156.2011.02828.x 21718394

[5] WHO consolidated guidelines on drug-resistant tuberculosis treatment. Geneva: World Health Organization; 2019. Available from: https://www.who.int/tb/publications/2019/consolidated-guidelines-drug-resistant-TB-treatment/en/ [cited 2019 Mar 4]. 30946559

[6] Interim guidelines: Updated recommendations on first-line and second-line antiretroviral regimens and post-exposure prophylaxis and recommendations on early infant diagnosis of HIV. Geneva: World Health Organization; 2018. Available from: https://apps.who.int/iris/bitstream/handle/10665/277395/WHO-CDS-HIV-18.51-eng.pdf [cited 2019 Mar 3].

[7] WHO advises on the use of multidisease testing devices for TB, HIV and hepatitis. Geneva: World Health Organization; 2017. Available from: https://www.who.int/hiv/mediacentre/news/multidisease-testing-hiv-tb-hepatitis/en/ [cited 2019 Mar 4].

[8] Waning B. Risks of decentralized procurement in fragile TB markets: Observations, implications, and recommendations at national and global levels. The Hague: Union Conference Presentation, Global Drug Facility and Stop TB Partnership; 27 October 2018. Available from: https://www.dropbox.com/s/kze0ojd8r0yo4qs/WANING_Domestic_Procurement_Public_27-Oct-2018.pdf?dl=0 [cited 2019 Mar 7].

[9] Step up the fight. Geneva: The Global Fund; 2019. Available from: https://www.theglobalfund.org/en/stepupthefight/ [cited 2019 Mar 4].

